# Identification and Analysis of Novel Tandem Repeats in the Cell Surface Proteins of Archaeal and Bacterial Genomes Using Computational Tools

**DOI:** 10.1002/cfg.358

**Published:** 2004-02

**Authors:** S. Adindla, K. K. Inampudi, K. Guruprasad, L. Guruprasad

**Affiliations:** 1 School of Chemistry University of Hyderabad Hyderabad 500 046 India; 2 Centre for Cellular and Molecular Biology Uppal Road Hyderabad 500 007 India

## Abstract

We have identified four novel repeats and two domains in cell surface proteins encoded by the *Methanosarcina acetivorans* genome and in some archaeal and bacterial genomes. The repeats correspond to a certain number of amino acid residues present in tandem in a protein sequence and each repeat is characterized by conserved sequence motifs. These correspond to: (a) a 42 amino acid (aa) residue RIVW repeat; (b) a 45 aa residue LGxL repeat; (c) a 42 aa residue LVIVD repeat; and (d) a 54 aa residue LGFP repeat. The domains correspond to a certain number of aa residues in a protein sequence that do not comprise internal repeats. These correspond to: (a) a 200 aa residue DNRLRE domain; and (b) a 70 aa residue PEGA domain. We discuss the occurrence of these repeats and domains in the different proteins and genomes analysed in this work.


**Comparative and Functional Genomics**
*Comp Funct Genom* 2004; **5**: 2–16.
Published online in Wiley InterScience (www.interscience.wiley.com). **DOI:** 10.1002/cfg.358

**Primary Research Paper**

### Identification and analysis of novel tandem repeats in the cell surface proteins of archaeal and bacterial genomes using computational tools

S. Adindla[1], K. K. Inampudi[1], K. Guruprasad[2] and L. Guruprasad[1]*
[1]*School of Chemistry, University of Hyderabad, Hyderabad, 500 046, India*
[2]*Centre for Cellular and Molecular Biology, Uppal Road, Hyderabad, 500 007, India*

**Correspondence to: L. Guruprasad, School of Chemistry, University of Hyderabad, Hyderabad 500 046, India. E-mail: lgpsc@uohyd.ernet.in*

Received: 30 May 2003
Revised: 21 October 2003
Accepted: 23 October 2003

### Abstract

**We have identified four novel repeats and two domains in cell surface proteins encoded by the *Methanosarcina acetivorans* genome and in some archaeal and bacterial genomes. The repeats correspond to a certain number of amino acid residues present in tandem in a protein sequence and each repeat is characterized by conserved sequence motifs. These correspond to: (a) a 42 amino acid (aa) residue RIVW repeat; (b) a 45 aa residue LGxL repeat; (c) a 42 aa residue LVIVD repeat; and (d) a 54 aa residue LGFP repeat. The domains correspond to a certain number of aa residues in a protein sequence that do not comprise internal repeats. These correspond to: (a) a 200 aa residue DNRLRE domain; and (b) a 70 aa residue PEGA domain. We discuss the occurrence of these repeats and domains in the different proteins and genomes analysed in this work. Copyright © 2004 John Wiley & Sons, Ltd.**

**Keywords:** **genome analysis; *Methanosarcina acetivorans* genome; cell surface proteins; tandem repeats; domains**

### Introduction

Most archaeal and eubacterial organisms possess a well-defined cell wall outside the plasma membrane (Beveridge and Graham, 1991). The cell wall present in Gram-positive and Gram-negative bacteria consists of a regularly ordered and planar array of proteins that make up the surface layer (Sleytr *et al*., 1996). The surface-layer proteins (SLPs) are associated with conservation and variation in their structure, biology and chemistry. The conserved properties are responsible for maintaining essential functions; such as mediating cell–cell interactions, transport, protection and virulence, whereas the variations may be a consequence of environmental/ecological pressures that mediate functions specific to that organism. In pathogenic bacteria, in addition to the functions mentioned, cell surface proteins are involved in various steps of infection processes, such as adhesion or invasion of host cells, binding to host molecules and protection against phagocytosis (Navarre and Schneewind, 1999). The surface layers are usually composed of high molecular weight glycoproteins that assemble spontaneously into two-dimensional crystalline arrays covering the entire cell surface (Beveridge, 1994). The SLPs have non-covalent binding and high-affinity interaction with the cell wall and make up to 15% of total protein content in prokaryotic cell (Mesnage *et al*., 2000).

Several SLPs are large, multi-gene proteins that consist of conserved domains. Some domains are common to various organisms. The SLP domains are of variable length (40–80 aa residues) with conserved sequence motifs that play a significant role in structure and function. In some SLPs, the domains are present as several copies and are responsible for anchoring to the cell wall or interaction with carbohydrates and lipids. The

Copyright © 2004 John Wiley & Sons, Ltd.

tandem repeats in SLPs identified to date are the SLH (surface layer homology), AB (also known as YVTN) and C repeats (also known as PKD).

The SLH domain is a repetitive modular element that is present in several bacterial cell surface proteins and is involved in non-covalent association with peptidoglycan-associated polymers (Lupas *et al.*, 1994). The SLH domain comprises 55 aa residues and the predicted secondary structure comprises two $\alpha$-helices flanking a short $\beta$-strand (Lupas, 1996). The AB repeats were first identified in bacterial SLPs of *Methanosarcina mazei* (Mayerhofer *et al.*, 1995). Recently, Adindla and Guruprasad (2003) identified AB repeats in several proteins that belong to various organisms, including the PE protein family in the *Mycobacterium tuberculosis* genome, and have predicted that its corresponding secondary structure comprises 4$\beta$-strands. The C-repeat, comprising 82 aa residues, also identified in bacterial SLPs (Mayerhofer *et al.*, 1995), is similar to the PKD (polycystin kidney disease) domain present in polycystin-1 and its solution structure has been determined (Bycroft *et al.*, 1999). Recently, Jing *et al.* (2002) determined the crystal structure of the N-terminal domain in *M. mazei* SLP (PDB Code: 1L0Q). This structure corresponds to the region encoded by YVTN repeats and a PKD domain. In fact, in the crystal structure, the YVTN repeat corresponds to four $\beta$-strands, as predicted (Adindla and Guruprasad, 2003). Further, the four $\beta$-strands adopt a $\beta$-propeller fold. General information about these repeats and domains may be retrieved from publicly available databases, such as INTERPRO (Mulder *et al.*, 2003), PFAM (Bateman *et al.*, 2002) and SMART (Letunic *et al.*, 2002).

In the present context, a 'domain' refers to a region of the protein sequence that does not contain internal sequence repeats. A domain can itself be repeated in a protein and there can be several different domains per protein. The domains identified in this manner may correspond to the generally accepted crystallographer's definition of a domain that represents a region of the protein capable of folding independently and is stable, e.g. signal transduction proteins contain SH2, SH3, PH domains. On the other hand, a 'repeat' corresponds to a region of the protein sequence that occurs more than once in tandem, e.g. the YVTN repeats in cell-surface proteins of *M. acetivorans*. Both repeats and domains can be characterized by 'sequence motifs' that may be identified according to the conservation of individual aa residues at equivalent positions derived from multiple sequence alignments.

Andrade *et al.* (2001) reviewed methods to identify repeating aa sequences in proteins and the relationship between repeat sequences and their associated functions. Repeats are thought to arise due to gene duplication and recombination events. While protein domains may exist either in high copy numbers or as a single copy per protein, repeats always exist as multiple copies (Andrade *et al.*, 2001, 2002). Repeats, often present in integer copy numbers, are usually associated with regular secondary structure and may vary in number indicating frequent loss or gain during evolution. When present in non-integer copy numbers, the first half of a repeat is present at the C-terminus while the second half is present at the N-terminus. This mode of circular permutation in repeats was proposed for the SLH domain in eubacterial proteins (Lupas, 1996).

Repeats may be identified by manual examination, if the sequence similarity is very high and if the repeats are present in tandem (Andrade *et al.*, 2001). Repeat boundaries are often difficult to predict; however, we show in this work that this can be achieved by examining the tandem repeats flanked by previously identified well-characterized domains and also by predicting their corresponding secondary structure. The popular web-based automated programs that identify internal repeats in proteins are REP (Andrade *et al.*, 2000) and RADAR (Heger and Holm, 2000). RADAR stands for rapid automatic detection and alignment of repeats in protein sequences. It uses an algorithm that segments the query sequence into repeats and identifies short, composition-biased, gapped approximate repeats and complex repeat architecture (Heger and Holm, 2000). Programs such as BLASTP (Altshul *et al.*, 1990) are also useful in detecting internal repeats and homologous repeats in a protein database. By using the BLAST program, the presence of repeats in a query protein sequence can be identified if: (a) the same region of the query is aligned against two or more distinct regions of a second protein; and (b) different regions of the query are being aligned against the same region of a second protein (Andrade *et al.*, 2001). When the PSI-BLAST program (Altshul *et al.*, 1997) is used, the statistically

Copyright © 2004 John Wiley & Sons, Ltd. 

significant repeats must be included in the profile for subsequent iterative searches. Once statistically significant repeats are detected, construction of a multiple sequence alignment provides insight into the extent of sequence homology among members of the new protein family and identification of the conserved sequence motifs.

Methanogenesis, the process of biological production of methane from acetate, is carried out by *Methanosarcina acetivorans* C2A. Methanosarcineae thrive in a wide range of environments and are unique amongst archaea in forming multicellular structures during different phases of growth and in response to environmental change. The complete genome sequence of *M. acetivorans* C2A was reported by Galagan *et al.* (2002); it is a model archaeal genome comprising 4524 open reading frames. A considerable portion of the *M. acetivorans* genome comprises multigene families. The large multigene families include several transport-related proteins and cell surface proteins. This organism synthesizes a cell envelope termed the S-layer, which consists of protein subunits adjacent to the cell membrane (Kandler and Konig, 1993). The majority of these proteins do not contain transmembrane regions and hence are secreted and play a role in generating the cell envelope (S-layer) as well as an extracellular matrix during the formation of multicellular structures (Galagan *et al.*, 2002). Major therapeutic and biotechnological applications may emerge from understanding the mechanisms underlying cell surface proteins in bacteria (Cossart and Jonquieres, 2000).

As the complete genome sequence of *M. acetivorans* is now available (Galagan *et al.*, 2002) and knowing that some cell surface proteins are associated with tandem repeats, we intended to systematically identify and analyse all sequence repeats in the cell surface proteins. We identified four novel tandem repeats. However, in the process we also identified two new domains. Further analysis corresponding to searches of the completed and unfinished genome databases also identified these repeats and domains in other archaeal and bacterial genomes.

### Methods

We extracted all cell surface proteins in the *M. acetivorans* genome by searching the SWall database in SRS (Schaftenaar *et al.*, 1996), available at **http://srs.ebi.ac.uk**. The keywords used were '*Methanosarcina acetivorans*' for organism and 'cell surface proteins' for all-text. All proteins thus retrieved were analysed with the RADAR program, available from **www.ebi.ac.uk/Radar/**. This program detects sequence repeats and generates multiple sequence alignments. No assumptions are made as to the expected length and number of repeats, and no restrictions are imposed on the sequence length that separates two or more repeats. In a typical analysis, a repeat region identified by the RADAR program was searched against the non-redundant GenBank and SWall databases using the PSI-BLAST and WU-BLAST2 programs, respectively. BLAST (Altschul *et al.*, 1990) is a reliable and rapid computer program that identifies in a given database all known proteins that are homologues/analogues to a query protein sequence. We also carried out similar searches against the completed and unfinished microbial genomes using the BLASTP program (**www.ncbi.nlm.nih.gov/BLAST/**). The Blosum62 matrices were used and hits were sorted on *p*-value in the WU-Blast2 program. The results of all BLAST searches were used for reciprocal searches once again in order to be able to retrieve the original query sequence. Sequences identified from the above searches were aligned using the multiple sequence alignment program CLUSTALW (Thompson *et al.*, 1994), available at **http://www.ebi.ac.uk/clustalw/index.html**. The default parameters used correspond to a penalty of 10 for opening a gap, 0.05 for gap extension and 8 for gap separation. The secondary structure predictions corresponding to aa sequences of either the repeats or the domains identified in this work were carried out using the PHD program, which uses the neural network method (Rost *et al.*, 1994) and is known to yield better than 70% prediction accuracy.

### Results and discussion

We identified 70 cell surface proteins (data not shown) in the *M. acetivorans* genome and only proteins containing sequence repeats were included in our analysis. We observed that several of these proteins correspond to the well-characterized

Copyright © 2004 John Wiley & Sons, Ltd. 

YVTN repeat (earlier referred to as the AB repeat), or to known domains, such as SLH and PKD. However, in this work, we identified four new repeats and two conserved domains in the *M. acetivorans* genome. The repeats or domains identified are not within (part of) previously reported repeats, such as the SLH, YVTN (or AB) repeats or the PKD domain. Our findings are therefore novel and may be characteristic of the cell-surface proteins. We also identified these novel repeats and domains in some of the proteins of other archaeal and bacterial genomes. The aa sequence patterns characteristic of these repeats and domains are represented according to the PROSITE description (Falquet *et al.*, 2002). The conserved aa residues inferred from multiple sequence alignments using the CLUSTALW program are used to describe sequence motifs characteristic of these repeats and domains. Often more than one sequence motif is associated with the tandem repeats or the domains. Lists of the proteins containing these repeats and domains are shown in Tables 1A–F. These tables give the protein identifiers (Gene or SWall), the number of aa residues in the protein, a description of the protein, and other well-characterized repeats and domains present in the protein, along with the number of such repeats or domains, including those identified in the present work. The schematic representations of repeats and domains analysed in this work are based on those of Galagan *et al.* (2002). The four novel repeats are labelled as; RIVW, LGxL, LVIVD and LGFP. The number of tandem repeats may vary within a protein. The two novel domains are labelled DNRLRE and PEGA. There can also be more than one copy of the domain in a protein. Some sequences representing these repeats or domains share lower than 15% pairwise sequence identity. However, we consider sequence pairs even with such low sequence identities if the corresponding e-values from the BLAST analysis are significant and if the individual sequences are characterized by the conserved sequence motifs, and if the PHD program predicts a similar secondary structure for the individual sequences.

The multiple sequence alignment program CLUSTALW is very useful for aligning representative sequences corresponding to a repeat from different proteins. However, owing to variation in the length of individual protein sequences and the number of repeats, CLUSTALW does not properly align the corresponding repeating sequence when the whole protein sequence is used in the multiple alignments. Therefore, these had to be edited manually and the resulting alignments reflect the aa conservation within individual repeats and in all repeats over the whole length of the protein sequence. The multiple sequence alignments and source sequences are available as an on-line supplement (**http://www3.interscience.wiley.com/cgi-bin/jabout/77002016/OtherResources.html**) and from our website at **http://202.41.85.161./-lgp/**. The aa sequences corresponding to the representative repeat in each protein and for all the proteins are shown in the multiple sequence alignments in Figures 1A–F. The schematic figures used to represent these repeats and domains are shown in Figures 2A–F. These figures (drawn to an approximate scale) reflect the relative proximity and location of individual repeats and domains along the sequence. We discuss each of these repeats and domains below.

### 42 aa residues RIVW repeat

The RADAR program identified aa sequence repeats, each corresponding to approximately 42 aa residues in several proteins. Seven repeats present in tandem were common to most proteins analysed. The database searches identified the repeats with significant scores (e-value $<10^{-6}$) also in proteins corresponding to other genomes. A list of proteins containing this repeat is shown in Table 1A. These include several hypothetical, predicted, conserved and surface layer proteins from *M. acetivorans, M. mazei* and *M. barkeri* (a genome sequencing project under way at the time of our present analysis). The aa sequence pattern corresponding to this repeat according to PROSITE notation is represented as [RK]-[IVL]-[VI]-[WY]. For the sake of simplicity, we refer to this as the RIVW repeat. The repeat boundaries, in this case, were assigned based on the identification of well-characterized neighbouring domains, e.g. in the protein corresponding to the gene identifier MA2706, we observed that the 909 aa residue cell surface protein contains three PKD domains sandwiched between two RIVW repeats. This is shown in the schematic representation in Figure 2A. Likewise, all 45 proteins listed in Table 1A containing the RIVW repeats may be associated with one of nine domain architectures (see Figure 2A). In the protein corresponding to gene identifier MM1677, the PKD domain

Copyright © 2004 John Wiley & Sons, Ltd.

is sandwiched between RIVW repeats and previously identified AB repeats. In the *M. mazei* protein corresponding to gene identifier MM2071 comprising 869 aa residues we observed that the RIVW repeats are associated with another 200 aa residue region referred to as the DNRLRE domain, which is discussed later. As can be seen from Figure 2A, there are three copies of this domain in MM2071. In another protein corresponding to the gene identifier MM2742 comprising 768 aa residues, we also identified another novel domain comprising 70 aa residues, referred to as the PEGA

**Table 1A.** Proteins containing the 42 amino acid residue RIVW repeat

| Gene or SWall identifier (No. of residues) | Organism | Description; other repeats or domains: No. of repeats or domains | No. of RIVW tandem repeats |
|---|---|---|---|
| MA2284 (1003) | *M. acetivorans* (A) | Cell surface protein; PKD:1 | 7 + 7 |
| MA2706 (909) | *M. acetivorans* (A) | Cell surface protein; PKD:3 | 7 + 7 |
| MM2630 (937) | *M. mazei* (A) | Conserved protein; PKD:2 | 7 |
| MA0487 (429) | *M. acetivorans* (A) | Predicted protein | 7 |
| MA1738 (970) | *M. acetivorans* (A) | Cell surface protein; PKD:2 | 7 |
| MM2923 (1164) | *M. mazei* (A) | Hypothetical protein; YVTN:7 | 7 |
| MM1677 (1063) | *M. mazei* (A) | Conserved protein; YVTN:7, PKD:1 | 7 |
| MA2794 (630) | *M. acetivorans* (A) | Hypothetical protein; PEGA:1 | 8 |
| MA1730 (411) | *M. acetivorans* (A) | Cell surface protein; PKD:1 | 2 + 5 |
| MM2296 (392) | *M. mazei* (A) | Conserved protein | 7 |
| MM2742 (768) | *M. mazei* (A) | Hypothetical protein; PEGA:2 | 7 |
| MA1293 (688) | *M. acetivorans* (A) | Cell surface protein; PKD:4 | 7 |
| MM2071 (869) | *M. mazei* (A) | Conserved protein; DNRLRE:3 | 7 |
| MA0488 (923) | *M. acetivorans* (A) | Cell surface protein; PKD:1 | 6 + 7 |
| MA0783 (345) | *M. acetivorans* (A) | Predicted protein | 7 |
| MM1936 (374) | *M. mazei* (A) | Conserved protein | 7 |
| MA2724 (380) | *M. acetivorans* (A) | Hypothetical protein; PKD:1 | 7 |
| MA2705 (330) | *M. acetivorans* (A) | Predicted protein | 7 |
| MA1838 (919) | *M. acetivorans* (A) | Cell surface protein; PKD:3, YVTN:7 | 5 + 2 |
| MA0484 (329) | *M. acetivorans* (A) | Predicted protein | 5 + 2 |
| MA0260 (328) | *M. acetivorans* (A) | Predicted protein | 5 + 2 |
| MM1670 (336) | *M. mazei* (A) | Conserved protein | 5 + 2 |
| ZP_00076576 (670) | *M. barkeri* (A) | Hypothetical protein; PKD:4 | 7 |
| ZP_00077009 (123) | *M. barkeri* (A) | Hypothetical protein | 3 |
| ZP_00076817 (581) | *M. barkeri* (A) | Hypothetical protein; PKD:3 | 7 |
| ZP_00076955 (678) | *M. barkeri* (A) | Hypothetical protein; PKD:4 | 7 |
| ZP_00077091 (938) | *M. barkeri* (A) | Hypothetical protein; PKD:3 | 7 + 7 |
| ZP_00076164 (161) | *M. barkeri* (A) | Hypothetical protein | 3 |
| ZP_00078197 (685) | *M. barkeri* (A) | Hypothetical protein; PKD:4 | 7 |
| ZP_00078648 (713) | *M. barkeri* (A) | Hypothetical protein; PKD:4 | 7 |
| ZP_00077008 (1001) | *M. barkeri* (A) | Hypothetical protein; PKD:3 | 7 |
| ZP_00077424 (728) | *M. barkeri* (A) | Hypothetical protein | 7 + 7 |
| ZP_00076578 (547) | *M. barkeri* (A) | Hypothetical protein; PKD:4 | 5 |
| ZP_00078647 (560) | *M. barkeri* (A) | Hypothetical protein; PKD:2 | 7 |
| ZP_00076954 (669) | *M. barkeri* (A) | Hypothetical protein; PKD:4 | 7 |
| ZP_00076999 (375) | *M. barkeri* (A) | Hypothetical protein | 7 |
| ZP_00075956 (231) | *M. barkeri* (A) | Hypothetical protein | 5 |
| ZP_00077721 (275) | *M. barkeri* (A) | Hypothetical protein | 6 |
| ZP_00075574 (149) | *M. barkeri* (A) | Hypothetical protein | 3 |
| ZP_00076982 (329) | *M. barkeri* (A) | Hypothetical protein | 5 + 2 |
| ZP_00076181 (754) | *M. barkeri* (A) | Hypothetical protein; PKD:3 | 4 |
| ZP_00077719 (819) | *M. barkeri* (A) | Hypothetical protein; PKD:2, YVTN:7 | 5 + 2 |
| ZP_00077090 (328) | *M. barkeri* (A) | Hypothetical protein | 7 |
| ZP_00077407 (771) | *M. barkeri* (A) | Hypothetical protein; PKD:2 | 7 |
| ZP_00077720 (713) | *M. barkeri* (A) | Hypothetical protein; PKD:4, YVTN:7 | 1 |

Copyright © 2004 John Wiley & Sons, Ltd.

**Table 1B.** Proteins containing the 200 amino acid residue DNRLRE domain

| Gene or SWall identifier (No. of residues) | Organism | Description; other repeats or domains: No. of other repeats or domains | No. of DNRLRE domains |
|---|---|---|---|
| MM1136 (1110) | *M. mazei* (A) | Conserved protein | 3 |
| Q977X0 (1077) | *M. mazei* (A) | Disaggregatase PbH1 : 7 | 3 |
| MM1144 (1095) | *M. mazei* (A) | Conserved protein | 3 |
| Q977Q4 (1077) | *M. mazei* (A) | Disaggregatase PbH1 : 7 | 3 |
| Q977X1 (1077) | *M. mazei* (A) | Disaggregatase PbH1 : 7 | 3 |
| MA0957 (1196) | *M. acetivorans* (A) | Hypothetical protein PKD : 1, LGxL : 7 S-layer-related duplication domain | 3 |
| MA2384 (1000) | *M. acetivorans* (A) | Predicted protein; PbH1 : 8, CADG:1 | 2 |
| MM2071 (869) | *M. mazei* (A) | Conserved protein; RIVW:7 | 1 + 2 |
| MM3280 (832) | *M. mazei* (A) | Conserved protein; PKD:1 | 1 |
| MM2946 (675) | *M. mazei* (A) | Hypothetical protein | 1 |
| MA1059 (981) | *M. acetivorans* (A) | Predicted protein; PKD:1, PbH1 : 9, CADG:1 | 2 |
| MA4442 (597) | *M. acetivorans* (A) | Hypothetical protein; PbH1 : 7, TonB_boxC:1 | 1 |
| MM1118 (640) | *M. mazei* (A) | Conserved protein; TonB_boxC:1 | 1 |
| MA3087 (936) | *M. acetivorans* (A) | Predicted protein; PbH1: 6 | 2 |
| MM2804 (723) | *M. mazei* (A) | Conserved protein | 1 |
| MM1120 (698) | *M. mazei* (A) | Conserved protein | 1 |
| MA4444 (699) | *M. acetivorans* (A) | Predicted protein; PbH1 : 8 | 1 |
| ZP_00078100 (889) | *M. barkeri* (A) | Hypothetical protein | 2 |

**Table 1C.** Proteins containing the 70 amino acid residue PEGA domain

| Gene or SWall identifier (No. of residues) | Organism | Description; other repeats or domain: No. of repeats or domains | No. of PEGA domains |
|---|---|---|---|
| MM 2742 (768) | *M. mazei* (A) | Hypothetical protein; RIVW:7 | 2 |
| MA2794 (630) | *M. acetivorans* (A) | Hypothetical protein; RIVW:8 | 1 |
| MA0637 (362) | *M. acetivorans.* (A) | S-layer-like protein | 2 |
| Q56436 (469) | *T. thermophilus* (B) | S-layer-like protein | 4 |
| TM0841 (456) | *T. maritima* (B) | S-layer-like protein | 4 |
| Q8RQ51 (353) | *L. interrogans serovarlai* (B) | S-layer like protein | 2 |
| AAN47834 (527) | *L. interrogans serovarlai* (B) | S-layer-like protein | 2 |

**Table 1D.** Proteins containing the 45 amino acid residue LGxL repeat

| Gene or SWall identifier (No. of residues) | Organism | Description; other repeats or domains: No. of other repeats or domains | Number of LGxL tandem repeats |
|---|---|---|---|
| MA0957 (1196) | *M. acetivorans* (A) | Hypothetical protein; PKD:1, DNRLRE:3 | 7 |
| ALL7024 (445) | *Anabaena sp* (B) | Hypothetical protein | 7 |
| CPN0799 (349) | *C. pneumoniae* (B) | Hypothetical protein | 7 |
| CPN0797 (365) | *C. pneumoniae* (B) | Hypothetical protein | 7 |
| CPJ0797 (365) | *C. pneumoniae* (B) | Hypothetical protein | 7 |
| CPN0798 (337) | *C. pneumoniae* (B) | Hypothetical protein | 7 |
| CPN1075 (674) | *C. pneumoniae* (B) | Hypothetical protein | 6 |
| CPN0796 (680) | *C. pneumoniae* (B) | Hypothetical protein | 6 |
| XF2069 (94) | *X. fastidiosa* (B) | Hypothetical protein | 2 |
| XF2349 (745) | *X. fastidiosa* (B) | Hypothetical protein | 7 |
| XF2021(200) | *X. fastidiosa* (B) | Hypothetical protein | 4 |
| XF1265 (309) | *X. fastidiosa* (B) | Hypothetical protein | 5 |
| ATU0778 (848) | *A. tumefaciens* (B) | Hypothetical protein | 4 + 2 |

Copyright © 2004 John Wiley & Sons, Ltd. 

**Table 1E.** Proteins containing the 42 amino acid residue LVIVD repeat

| Gene or SWall identifier (No. of residues) | Organism | Description; other repeats or domains: No. of repeats or domains | No. of LVIVD tandem repeats |
|---|---|---|---|
| MA1510 (2115) | *M. acetivorans* (A) | Hypothetical protein; PKD:1, LGFP: 8 | 7 |
| MM 0391 (761) | *M. mazei* (A) | Hypothetical protein; PKD:1 | 10 + 4 |
| MA4 034 (757) | *M. acetivorans* (A) | Hypothetical protein; PKD:1 | 10 + 4 |
| TM0946 (316) | *T. maritima* (B) | Hypothetical protein | 6 |
| ZP_00077673 (1970) | *M. barkeri* (A) | Hypothetical protein | 4 + 10 |
| ZP_00065207 (14609) | *M.r degradans* (B) | Hypothetical protein | 2 + 2 |
| ZP_00045566 (11699) | *Magnetococcus* sp. MC-1 (B) | Hypothetical protein | 2 |
| ZP_00099871 (373) | *D. hafniense* (B) | Hypothetical protein | 6 (not tandem) |

**Table 1F.** Proteins containing the 55 amino acid residue LGFP repeat

| Gene or SWall identifier (No. of residues) | Organism | Description; other repeats or domains: No. of repeats or domains | No. of LGFP tandem repeats |
|---|---|---|---|
| CGL2875 (657) | *C. glutamicum* (B) | PS1 protein precursor | 5 |
| CGL1840 (527) | *C. glutamicum* (B) | Hypothetical protein | 4 |
| CGL1848 (629) | *C. glutamicum* (B) | Hypothetical protein | 5 |
| CGL1890 (528) | *C. glutamicum* (B) | Hypothetical protein | 5 |
| CGL0794 (527) | *C. glutamicum* (B) | Hypothetical protein | 4 |
| CGL2546 (540) | *C. glutamicum* (B) | Hypothetical protein | 5 |
| RV2721 (699) | *M. tuberculosis* (B) | Hypothetical protein | 6 |
| ML1002 (687) | *M. leprae* (B) | U22351 (Possible conserved membrane protein) | 6 |
| RV3811 (539) | *M. tuberculosis* (B) | CSP | 2 |
| Q9KIJ0 (246) | *M. paratuberculosis* (B) | Rv2721c-like protein | 2 |
| MA1510 (2115) | *M. acetivorans* (A) | Hypothetical protein; PKD:1, LVIVD:7 | 8 |
| DR1115 (398) | *D. radiodurans* (B) | S-layer-like array-related protein | 3 |
| CE2709 (669) | *C. efficiens* (B) | PS1 protein | 5 |

The proteins are represented by their corresponding gene or SWall identifiers along with the number of amino acid residues indicated in brackets in the first column. The organism and corresponding phylogeny are indicated in the second column; 'A' represents archaea and 'B' represents bacteria, respectively. The third column contains the description of the proteins containing the repeats or the domains identified elsewhere, including those identified in the present work and the total number of such repeats or domains. The fourth column represents exclusively the total number of novel tandem repeats or the domains observed in this work, in proteins represented by their corresponding gene or Swall identifier in the first column. In Table 1A, PKD, PEGA, DNRLRE represent domains (the latter two identified in this work) and YVTN is a tandem repeat. Proteins corresponding to gene identifiers MM1677 and MA1838 are also associated with the EF_hand motif that is known to bind calcium. The fourth column, e.g. indicating 7 + 7, represents two distinct regions along the protein, each corresponding to the seven RIVW tandem repeats, and so on. In Table 1B, CADG, TonB_boxC represent domains and PbH1 is a repeat. In Table 1C, S-layer like protein is a domain conserved in some surface layer proteins. In Table 1D, the gene identifier MA0957 comprises a S-layer related domain. In Table 1E, the gene identifier ZP_00077673 comprises YD repeats. In Table 1F, the gene identifier RV3811 comprises a peptidoglycan recognition protein (PGRP) region.

domain, which is discussed later. Table 1A shows that proteins containing RIVW repeats may have variable numbers of individual repeats with the exception observed in the protein corresponding to gene identifier ZP_00077720 that is identified with a single RIVW copy. Further, in (cell surface) protein corresponding to gene identifier MA1838 comprising the RIVW repeats, there are intervening aa residues between the fifth and sixth repeats. We observed that sequences corresponding to the RIVW repeat containing proteins shown in Table 1A have pairwise percentage sequence identities that vary (range 9–73%). The consensus secondary structure is predicted to comprise four $\beta$-strands (see Figure 1A). The tandem AB repeats associated with other cell surface proteins

Copyright © 2004 John Wiley & Sons, Ltd.

are known to form a $\beta$-propeller (Jing *et al.*, 2002). Likewise, it is possible that the RIVW repeats identified by us in cell surface proteins and predicted to comprise 4$\beta$-strands in each repeat may also form a $\beta$-propeller (as represented in Figure 2A) although we have not carried out analysis to verify this in the present work. Further, we observed that the repeats are specific to the genus *Methanosarcina*, as shown in Table 1a. This suggests that these proteins may form a specific array on the cell surface and mediate a function specific to this genus via the possible $\beta$-propeller structure.

### 200 aa residues DNRLRE domain

In the protein represented by the gene identifier MM2071, we identified a 200 aa residue region in addition to the RIVW repeat (see Figure 2A). This region is referred as a domain as it does not comprise internal sequence repeats. The domains are present towards the N and C-termini in MM2071. The extent of similarity shared between the domains is greater than 65%. Further searches of the databases using the sequence corresponding to this domain (position 472–671) as a query in the BLAST program, we identified several proteins

(a)
```
Secondary structure          eeeeee          eeee eeeeee                eeeeeee
MA2706(611-651)           ENEIRITTSELASY---PDIYGNRIVWQDLR--NG-------NYDIYMYDLST 41
ZP_00077009(21-66)        SEKIQISTSGLAFD---PSIYGNRIVWRDSR--NGKEYI--ENSNIYMYDLST 46
ZP_00076578(7-47)         KIQTRISKSGEAHN---PAIYGNRIVWQEES--NG-------NSNIYMYDIST 41
MM2742(182-225)           TKETQITTNGSASEYGSPAIYGDRIVWQDERDG---------NSDIYMYNLST 44
MA1730(284-324)           ANETRISTNGSAS---SPAIYGNRIVWQDRRTN---------QSAIYMYDLSA 41
ZP_00076817(36-76)        ITETRITNHGTAS---NPDIYGDKIVWQDNRNG---------NWDIYIFDLST 41
MM2296(164-207)           KKETRITTNGSAA---IPSIYGDKIVYHDWRNGFLK------YSDIYMYDLST 44
ZP_00076999(61-99)        VTETQITTSG--FH---PAIYGNRIVWTDYH---------DEECNIYMYDLST 39
ZP_00078197(40-85)        INETPITTSGSATS-- PSIYGDRIVWKDWRNGNRD----DGPFNIYMYNIST 46
ZP_00076576(77-125)       QKETQITTSGSATS---PSIYGNRIIWLDGRNGNSQNDT-EGGHDVYMYDLST 49
ZP_00077091(829-870)      SKKTQITTNKSSQY--YPAIYGNKIVWEDFR--NEYIN-------IYMYDLST 42
ZP_00076181(656-699)      SKQARITNNKSSSY--LPAVYGNRIVWESRRIANGSSN-------IFTYDLST 44
ZP_00077720(17-63)        DKEIQITNDESDQL--NPAIYGDKIVWEDYR--NDRENM-YYCS-IYMYDFSA 47
ZP_00075574(42-88)        KKETQITSSLDDQT--SPDIYGDKIVWEED---GGKDAV-YTNHGIYMYDIST 47
MA0484(106-143)           ATKSYITQNVDQFS--KPAIYGNRIVWSADD--N-----------VYLWDIST 38
ZP_00077407(106-150)      TAKTYITQNVDQYS--RPVIYENRIVWSADY--NESN----YNYNVYMRDIST 45
ZP_00076955(83-124)       KKETRITTNTSDKW--DPAIYGNRIVWVDDR--NRS-------WDIYMYDLST 42
ZP_00078647(121-169)      KKETQITTNVSDQY--SPYIYGNRVVWVDER--NRNPEDFSGNSDIYMYDLST 49
MA1738(782-823)           TKETQITINNGFLE--DFAIYGDRMVWVDDR--NRN-------ADIYMYDLST 42
ZP_00076954(203-244)      KKETQITNGSSWAI--DPAIYGDRIVWMDER--SGN-------YDIYMYDLST 42
MA0783(130-171)           KKETQITTDKADQS--QPAISGNIVVWKDTR--SG-------NDDIYMYDIST 42
MM1936(160-201)           KEETRITDDKADQS--QPAVWGNIIVWKDTR--NG-------NEDIYMYDISA 42
MA2794(145-190)           QSETQITTNKSDQK--SPDIYGNRVVWEDDR--NGGD---LINSDIYMYDLST 46
ZP_00077424(73-114)       STETRITTNESAQS--GPVIYGNRIVWYDAR--NGNG-------DIYMYDLST 42
ZP_00078648(83-124)       ITEKRITTNNFEQS--DPVIYGNKILWYDER--NGNG-------DIYMYDLST 42
MM2630(663-708)           SKETRITTNESYQG--DPSIYGDKVVWQDSR--NGDGYN---PADIYMYDLST 46
MA2284(109-150)           SREVQITTSESYQG--KPAIYGDKIVWEDDR--NGNR-------DIYMYDIST 42
MM2923(442-483)           STEFQITTGESCQI--NPAIYGNKIVWQDDR--GGKS-------DIYMYDLST 42
MM1677(142-183)           SKETQITTNGSSQA--LPAIYGDKIVWQDQR--NG-------NWDIYMYDLSN 42
ZP_00077721(235-275)      SLETQITFNGSRKD--KLAIYGDRIVWQDDR--NG-------NWDVYVYDLC- 41
MA1293(239-280)           SAETQVTTDGLSHS--ISAIYGDRIVWEDNR--NG-------NWDIYMYDLST 42
MA0487(269-312)           SAESQITTSGSIES--GSAIYGNWIVWMDYR--KGWE-----NLDVYLYDLST 44
ZP_00077008(610-651)      LTEYQITTNGVNPS--RPAIYKDRIVWSDNR--NG-------NPDIYTYDLST 42
MA0488(20-61)             SKEIKITTNGSSKA--NSAIYGNRVVWIDYR--TGNS-------DIYMYDIST 42
MA2724(209-252)           NKTTQVTSNVSAYQ---PSIYGNWIVYMIG-DPYSGGN-----KDIYMYEIPA 44
ZP_00075956(141-184)      HKVTQVTNSGNAID---PAIYGKRIVYTLN-RPHSFGV-----GDIYMYEMST 44
MA2705(240-284)           HETRQVTFDGNSTS---PDVYGDRIVWESAYRAGNNPS-----GDIYMYNILT 45
ZP_00076164(40-83)        TASGCVHENGCVYEN-EPKIFDDKIVWGER---YSNSG-----NNIYMYDFYT 44
ZP_00077719(66-105)       KTDTTVSSSAASH----PAIYGNVVVWHDES--NGMP-------RLTVYDIKT 40
MA1838(66-105)            KTDTTVSSSAASH----PAIYGNKLVWHDKS--SGVP-------RLTVYDIPS 40
ZP_00076982(67-105)       RMDTTVSSSGAFS----PDIYGNTLVWRDER--SGTP-------KLAVYDIPT 40
MM1670(66-105)            RTGIRFSSSGASS----PDIYENKIVWHDES--IGTP-------RIAVYDIPT 40
MA0260(197-237)           KKSIDVSQYGDNMF---SHIYGDKVIWSDFY--TRLG-------NIRMYDLAT 41
ZP_00077090(200-240)      KRVSDISRSGRAGN---GKIYGNIVVWTENQ--NGSR-------YVYMRDIAK 41
MM2071(436-479)           SKETQITTSGNAMY---PDIYGDTIVWIDHEDIYTDK------NDIFVGTVSE 44

consensus/80%             ppctplosstst.....PsIYGs+lVW.-tp..tt........plYhYDluo
```

**Figure 1.** The multiple sequence alignments corresponding to representative repeats and domains from various proteins along with their gene or SWall identifiers and secondary structure predictions (e, strand; h, helix) for (A) RIVW repeat, (B) DNRLRE domain, (C) PEGA domain, (D) LGxL repeat, (E) LVIVD repeat and (F) LGFP repeat. The numbers given in brackets indicate the start and end amino acid residue positions corresponding to either the repeat or the domain. The 80% consensus is labelled according to the alignment generated at the website **www.bork.embl-heidelberg.de/Alignment/consensus.html**: alcohol (o, ST); aliphatic (l, ILV); any (., ACDEFGHIKLMNPQRSTVWY); aromatic (a, FHWY); charged (c, DEHKR); hydrophobic (h, ACFGHIKLMRTVWY); negative (−, DE); polar (p, CDEHKNQRST); positive(+, HKR); small (s, ACDGNPSTV); tiny (u, AGS); turn-like (t, ACDEGHKNQRST). A capital letter indicates 80% conservation of corresponding amino acid residue. The secondary structure prediction indicated at the top was derived using the PHD program. Residues forming $\beta$-sheets are represented by 'e' and residues forming $\alpha$-helices is given by 'h'

Copyright © 2004 John Wiley & Sons, Ltd.

(b)

```
Secondary structure         eee             eeee          eeee eeee            eeeeeeee
MM1118(437-636)           VVETDKTSLTEENSS-DIIDNRLKESTPDTVYQDKEYLDIGGRPGIGKYRDLLLFNLSEY
MA4442(391-591)           VAGTENTSSTEENATGIVIDNRLREASPDVVYQDKVYIDIGGRPGVGRYRDLILFDLSKY
MA3087(514-714)           TETEENVSFTEENATGIIADNRLREASPYITYQEKAYIDIGGRPGVGRYRDLILFDLSKY
Q977X0(664-863)           TTKETPAPIIINETINEAIDNRLREASPDSVYQDSAFIDVGGMN-DARYRDVIWFDLDEF
MM1144(682-881)           TTKETPAPIIINETINEAIDNRLREASPDSVYQDSAFIDVGGMN-DARYRDVIWFDLDEF
Q977X1(664-863)           TTKETPAPIIINETINEAIDNRLREASPDSVYQDSAFIDVGGMN-DARYRDVIWFDLDEF
Q977Q4(664-863)           TTKETPAPIIINETINEAIDNRLREASPDSVYQDSAFIDVGGMN-DARYRDVIWFDLGEF
MM1136(693-896)           AEKALPVPVTIDKTITRAIDNRLREGSPDTVYQDSSFIDVGGMN-DARYRDVMWFDLSVY
MA0957(598-798)           VSVTPPEDSEEIVSEIEVSDNRLREASPDTVYKSSSFIDVGSISGVGRYRDAIQFDLSEY
MM2071(472-671)           IFVGTVSEGKYIISEIGVSDNRLREASPDTVYKDSSFIDVGGMN-NVRYRDILQFDLSKY
MA2384(593-792)           KQEDTSKQEDPSTITGEVYDNRLREASPDIVYQSSPFIDVGAMS-IGSYRDIMWFDLSVY
MM3280(630-829)           TPPEDSISDGSAASKLKVFDNRLREASPDTVFQSSPYIDIGGMN-SVRYRDMVWFNLSEY
MM2946(474-673)           TKYASKSGSAGDQAAGKVYDNRLREASPEAVFQNTSFIDIGGMS-TGRYRDAMWFDLSKY
ZP_00078100(688-886)      PKLNIEKRVTANATITDAKDNRLREISPEGVFSDTPFIDAGELSNVGKYRDVISFNLSEY
MA1059(584-781)           ANITVVENDSNPDEDIKIYDNRLREASPDTVIQNKPFIDVGGTDNVGRYRDVMWFNLSEY
MM2804(521-720)           SPGLQTSAPTAGPQISEMYDNRLREKSPEYTYPSKPCLDLGNSPGVGNDRDIIWFDLSGY
MM1120(495-693)           ISGESDNEELEIVLPLVISDNRLKEENPDSTLRDTEYIDVGESPDGGKYRGVILFELGQL
MA4444(496-694)           ASGESEEENLKIISLSVASDNRLKEEAPNTTYRETEYIDVGERPGGGIYRDVMLFELKQL
ZP_00077909(493-692)      SMQSDKAENLKIALPFIISDNRLREEAPNITFSDSEYIDVGKKSDGGIYRGVIIFDLSSL

consensus/80%             s..t..t..h...t...h.DNRLREtoP-.sapsp.aIDlGths.supYRDlhhFsLspa

Secondary structure                 hhhhh                eeeeee         eeeee     eeee
MM1118(437-636)           ND----AENISNATLSLYWYYPDGIERPEDTIVEVYRPAAAWNPENVTWNTRDNGVLWTQ
MA4442(391-591)           DE----AENITNATLSLYWYYPDGIERPEDTIVEIYRPAAAWSPENVTWNSRDTGVLWTQ
MA3087(514-714)           DD----AENITSATLSLYWYYPDGRARPEDAIVEIYRPAAAWDPQSVTWNSRGDCVPWTY
Q977X0(664-863)           ND----TTEVTDSTLSLYWYYPAGNERPDDTVIEVYRPASEWNSSYVNWNKKDKNVAWKN
MM1144(682-881)           ND----TTEVTDSTLSLYWYYPAGNERPDDTVIEVYRPASEWNSSYVNWNKKDKNVAWKN
Q977X1(664-863)           ND----TTEVTDSTLSLYWYYPAGNERPDDTVIEVYRPASEWNSSYVNWNKKDKNVAWKN
Q977Q4(664-863)           ND----TTEVTDSTLSLYWYYPAGNERPDDTVIEVYRPASEWNSSYVNWNKKDKNVAWKN
MM1136(693-896)           DETAEVSTEVTGATLSLYWYYPAGNTRPDDTIVEVYRPASSWNTSYVSWNKRDKNVAWKN
MA0957(598-798)           NS----DSQITNAVLSLYWYYPSGTTRPEDTVIEIYRPASAWNSSYVSWNKRDKNVAWTN
MM2071(472-671)           TS----NSRITNAVLLLYWYYPAGKTRPEDTVIEIYRPASSWNLDYVSWNKKDKNVAWEK
MA2384(593-792)           AD----YSEVNSATLSLYWYYPAGKARPEDTVIEIYRPADSWNPDYVSWNKKDKRVAWNN
MM3280(630-829)           TG----SANVNNATLSLYWYYPAGISRPSDTVIEVYRPASSWNPGYVSWNKRDRGIAWKN
MM2946(474-673)           ET----SAEIDNATLSLYWYYPAGKTRPEDTVIEVYRPASAWNPDYVSWNKRDRGIAWKN
ZP_00078100(688-886)      TS----ATEVDSATLSLFWYYPSS-TRSNDTVIEIYRPVS-WNPDYVSWNKKNKDIAWNN
MA1059(584-781)           SD-----QKISKAIISLYWYYPEE-SRPEDTVIEVYRPAS-WNPSYVSWNNRDNGVKWTN
MM2804(521-720)           AD----AEKISSAVLSLYWYYPTVPKTRD-TVVDLYRPADSWDPDHVSWNKKDRGVKWKN
MM1120(495-693)           NKT----NKIEKATLSLFWYYPE-ESRKEDTILEVYRPEK-WCEEHISWGAREINTPWKN
MA4444(496-694)           DET----DSIEKATLSLFWYYPE-EARPEDIVLEVYRPEK-WCEEHVTWEEREIETPWQN
ZP_00077909(493-692)      NQT----DQVDEATLSLFWYYPENQIRSKDTILEVYRPTK-WCREHVTWQQRESNDPWKN

consensus/80%             sp.....pplspusLSLaWYYPts.tRs-DTllElYRPss.Ws.paVsWNp+-psl.Wpp

Secondary structure            eeeeeeeee     eeeeeeee       eeee hhhhhhh     eee   eeeeee
MM1118(437-636)           PGGDWFDMNNVSQGDAPYATITIKGSDIPDNRYYELNVTDLVKEYVSGEYENTGFLIKTR
MA4442(391-591)           PGGDWFDMNNTSQGDAPYATITLKGSDIPDNRYYELNVTELVKEYVSGEYENTGFLIKTQ
MA3087(514-714)           PGGDWFDKDGILQGDDPYATITLKGCSLPDNRYYEINVTELVKEYASGKYENTGFLVKTR
Q977X0(664-863)           AGGDWYDKNGITQGDTPYASIALKGSELPDNKYHEIDVTELVNEYVSGKYENTGFLIKAR
MM1144(682-881)           AGGDWYDKNGITQGDTPYASIALKGSELPDNKYHEIDVTELVNEYVSGKYENTGFLIKAR
Q977X1(664-863)           AGGDWYDKNGITQGDTPYASIALKGSELPDNKYHEIDVTELVNEYVSGKYENTGFLIKAR
Q977Q4(664-863)           AGGDWHDKNGATQGDTPYASIALKGSELPDNRYYELDVTELVKEYTSGKYENTGFLIKAR
MM1136(693-896)           AGGDWYDKNGVLQGSTPYATFTIRGSAFPDNRYYELDVTELVKEYTSGKYENTGFLIKAR
MA0957(598-798)           EGGDWYDRNGVLQGSTPYATITIKGSSLPDNRYYELDVTDLVKEYVSGKYENTGLLIKAR
MM2071(472-671)           PGGDWYDKNGILQGNTPYATITVKGSFPPGDKYYELDVTDLVKEYTSGKYENTGFLIKAR
MA2384(593-792)           AGGDWYDRNGVLQGSTPYATITLEGSDLPDNRYYELDVTDLVNEYISSKYENTGFLIKAR
MM3280(630-829)           PGGDWYDKKGVLQGSTPYATLTIKGSALPDNRYYELNVTDLVKEYVSGKYENTGFLIKAR
MM2946(474-673)           PGGDWYDKNGVLQGSTPYATVTLKSSTLPDNKYYKLDVTDLINEYIGGKYVNTGFLIKAR
ZP_00078100(688-886)      AGGDWYDRNGVLQGSTPYATLTLRASSLPDNRYYELNVTDLVKEYVSGRYENTGFLIKAR
MA1059(584-781)           AGGDWYDKEGVSQGNTPYAKFTIKGKELPDNRYYELDITDLVKEYVSGEYENTGFLIKAR
MM2804(521-720)           AGGDWYDKKGAAQGKTPYATFTIKSGTLPDFRYYELDVTDLVKEYVSGKYENTGFLIKTR
MM1120(495-693)           PGGDWYDKNGVSQGSTPYSTITFKSSSLPDNRYYELDVTELVREYISGKYENTGFLIKSR
MA4444(496-694)           PGGDWYDRNDVLQGSMPYATITIKGSTLPDNRYYELDVTELVKEYVSGEYENTGFLIKAR
ZP_00077909(493-692)      SGGDWYDRNGIFQGSTPYAKVTISGDETPDNRYYDLDVTELVQEYISGKYENTGFLIKAH

consensus/80%              GGDWaD+sGl.QGssPYAolsl+uuplPDN+YYElsVT-LVpEYhSGcYENTGFLIKsR

Secondary structure       e     eeeeeee         eee
MM1118(437-636)           AENADYVAFYSSEIDDENQRPMLAI 200
MA4442(391-591)           NENADYVAFYSSDIEDKDKRPTLNI 201
MA3087(514-714)           TEDADYIAFYSNEGGNESQRPRLNV 201
Q977X0(664-863)           DENNNYIAFYSNECGKETQKPSLNI 200
MM1144(682-881)           DENNNYIAFYSNECGKETQKPSLNI 200
Q977X1(664-863)           NENNNYIAFYSNECGKETQKPSLNI 200
Q977Q4(664-863)           NENNNYIAFYSNECGKETQKPSLNI 200
MM1136(693-896)           NENNNYIAFYSNECGKETQKPSLNI 204
MA0957(598-798)           TESNNYIAFYSSDWTDENQKPKITV 201
MM2071(472-671)           TDSNNYIAFYSSDWTDENQKPKITV 200
MA2384(593-792)           TENNNYIAFYSNDCGNENQKPKLTV 200
MM3280(630-829)           TERNNYIAFYSSDCGNENQIPKIRL 200
MM2946(474-673)           TENNNYIAFYSMEAGSENQRPKLDL 200
ZP_00078100(688-886)      SENDNYIAFYSADCGNMNQVPKLNL 199
MA1059(584-781)           SESNNYIAFYSSDCGNESQEPKLKI 198
MM2804(521-720)           YENSDYVAFYSLECDEGDFEPKLDI 200
MM1120(495-693)           AEDSDYIAFYSSEWQNKNQMPRLSI 199
MA4444(496-694)           SESSNYIAFYSSEWQNKAQRPKLTI 199
ZP_00077909(493-692)      EEDENYVAFYSSNWQNKNQRPKLTI 200

consensus/80%             sEsssYlAFYSs-htpcsQcPpLsl
```

**Figure 1.** Continued

Copyright © 2004 John Wiley & Sons, Ltd. 

```
(c) Secondary structure           eee    eeeeee eee          eeee   eeeeeeee  eeeeeeeeeee
    Q56436(187-256)        EATLEVDSSPRGAEVYVDGRREGKTP---LSLAVRPGRHEVELRLPGYAPYRAAVNARPG
    TM0841(314-386)        QSSLKLRTDPSGVDVYIDGRYVGTTDQNGLNLILDPGMYEVKLEKEGYETDRFTVNLAPG
    MM2742(571-643)        FKKLRITSIPEGANILLDGEYIGKTP--KETKITDLRTYLICLELEGYERWEQKSEVQAS
    MA2794(562-630)        STDLRISSIPEGAKASIDGKYIGKTP--KSISIGELKTYSVQLELEGYKNWNGQCKFDKL
    Q8RQ51(118-188)        DGLISVTSNPEGASVYLGSEFLGKTP--ITNVRVKTGYNRLRLSMEGHVDLLKGVEIKKD
    AAN47834(292-362)      DGLISVTSNPEGASVYLGSEFLGKTP--ITNVRVKTGYNRLRLSMEGHVDLLKGVEIKKD
    MA0637(133-202)        RWTYSVSSSPSGAKVYLDEGYKGVTP---VVFNAEGRQHKLTIKKTGYGTVSKEINASDD

    consensus/80%          pt.lplsS.PpGAplhlsucahGpTP....shhsc.th.plpLphpGa.sh.ttsphp.s

    Secondary structure     eeeeeeee
    Q56436(187-256)        ERVRVFAR--LVPEP 70
    TM0841(314-386)        EEKEIFRR--LEKRV 73
    MM2742(571-643)        NKSEVQVEALLTEKQ 73
    MA2794(562-630)        EKQEIQPT--LSR-- 69
    Q8RQ51(118-188)        EETKLDLV--LKQGN 71
    AAN47834(292-362)      EETKLDLV--LKQGN 71
    MA0637(133-202)        PSILIEEK--LHLSL 70

    consensus/80%          pc.cl..h..Lp.t.
```

```
(d) Secondary structure              eeeee       eeeeeee    eeeeeee        eeeeee
    ALL7024(265-312)    -NSSINPNTDDLGTLGGS---YSEAKAINNLGQ-VVGFSTTANGE---TNAFLTAP 48
    MA0957(222-264)     --VTIT----DLGTLGGN---YSNAEGINNKGQ-VVGTSQTDTGV---EHAFLWQN 43
    CPJ0797(95-138)     --HLIK----HLGTLGGE---ASSAEGISKDGEVVVGWSDTREGY---THAFVFDG 44
    CPN0797(95-138)     --HLIK----HLGTLGGE---ASSAEGISKDGEVVVGWSDTREGY---THAFVFDG 44
    CPN0796(333-376)    --GQMV----DLGTLGGP---ESYAQGVSGDGKVIVGRAQVPSGD---WHAFLCPF 44
    CP1075(327-370)     --GQMV----DLGTLGGP---ESYAQGVSGDGKVIVGRAQVPSGD---WHAFLCPF 44
    CPN0798(293-336)    --GRMI----DLGTLGGS---ASFAFGVSDDGKTIVGKFETELGE---CHAFIYLD 44
    CPN0799(274-317)    --GVMS----DLGTLGGS---YSAAKGVSATGKVIVGMSTTANGK---LHAFKYVG 44
    XF2021(75-124)      -ENWET--KTRLGSLRSDNLGNSKVVALSANGKIAAGYSETDSKT---IHAVIWSG 50
    XF1265(91-143)      -DNWAT--KTELGSLKSDSSGASIVVALSSDGKIAAGQSSIDSRYSNLREATIWSG 53
    XF2069(43-92)       -KNWAT--KTDLGTLQKDNLGSSYVTALSSDGKIAVGYAETDSKS---LHAIIWSG 50
    XF2349(375-424)     -DHWQT--KIDLGTLKSDNSGYSISTALSADGTVAAGYSEVDSGK---DHATVWKI 50
    ATU0788(208-253)    ATGVMT----AIDMPADVT--SSVANDVSLDGRVVVGEYFTAANV----HAFRWTA 46

    consensus/80%       ..t.hh....cLGoLtus....S.s.ulStsGphhsGhupstpt.....HAhlh..
```

```
(e) Secondary Structure              eeeeeee     eeeee       eeeee       eeeee
    ZP_00065207(3352-3393)  NGFTRDVKIAGRYAYIAAS--HEGVVVADVADPSMPIIAKIDTL 42
    MA1510(170-210)         SGDAWDVAVSGKYAYVAFG--AG-LVIVDISAPTSPTLVGSYDT 42
    ZP_00077673(378-419)    SGTTYAVAVSGNYAYLASG--DNKLVIVDISNLSSLKFASSCYT 42
    MM0391(415-456)         GGWAQGITVSGNYAYVIDM--ANGIFIVDISNPSSPILEGMYDT 42
    MA4034(414-455)         GGWAQHITVSGNYAYVTDN--ANGIFIVDIGNPSSPTLKGIYDT 42
    ZP_00045566(7290-7329)  MGAPNGIAVSGQTAYIS----QGDLLAINISNPTAPSVIGVYDE 40
    TM0946(263-300)         GGKAQSLWLYEGFLYIADF--NGYLTVVDVSDPSHMNEVFNVLT 42
    ZP_00099871(14-58)      HGKTMQVMKYKDYLYVGNMVPGIGTIIVDVTNPSLPLVCGEMPA 44

    consensus/80%           tGhs.tlhl.tpahYls....tt.lhllDlusPo...hht.h.t
```

```
(f) Secondary Structure                    eeeee  eeee         hhhhhhhh
    RV2721(170-221)      LGAPVGDET--YDGEVTAQKFSGGEVSWNRATKEFTTVPAVLAEQLKGLQVAID- 52
    ML1002(163-214)      LGVPVADES--FDGEVISQKFSGGAVFWNKKSSEFTTEPTALAEQLTGLLVATD- 52
    CGL1848(168-221)     LGPPKSNELTNPDGVGKRSEFVGGAIYWHPDTGAYA-VTLDGLRQWGTLNWESGP 54
    CGL1890(67-120)      LGPPKSNELTNPDGVGKRSEFFGGAIYWHPDTGAYA-VTLDGLRQWGTLNWESGP 54
    CGL2875(464-517)     LGYPTSSELKTPDGRGRFVTFEHGSIYWTATTGPWE-IPGDMLAAWGTQDYEKGS 54
    CE2709(476-529)      LGFPKTRELSTPDGRGRYVHFENGSIYWSAATGPWE-IPGDMFTAWGTQGYEAGG 54
    RV3811(420-472)      LGAPTSPEADAADG-ARYATFAKGAMYWSPVTDAQP-ITGAIYEAWASQSYERGP 53
    CGL1840(278-331)     LGFPIADEAVTADGVGRFSVFQNGVVYWHPQHGAHP-ILGDIYSIWREEGAESGE 54
    CGL0794(287-331)     LGFPIADEAVTADGVGRFSVFQNGVVYWHPQHGAHP-ILGNIYSIWREEGAESGE 54
    CGL2546(276-329)     LGFPIADEAVASDGVGRFSVFQNGVLYWHPNHGAWE-MTGFIEEVWKMRGGLDSQ 54
    Q9KIJ0(157-207)      LGAPTGNEQKNPDG-GVYQQFDGGVI--VSKTQAYV-VWGKIRDKWNQLGGSQGQ 51
    MA1510(603-654)      LGFPITDQR-EKDG-HDYCVFEGGIIDWNDSTGTYDVKLGYEGLLFRAKDGIDV- 52
    DR1115(239-290)      LGDPTIYATRWADG--WWQRFE-GVGAYGDAVLLHANGSSRAYAVHGAIFKRYLD 52

    consensus/80%        LG.PhssEh...DG.shht.FttGsl.Wpstpt.a..h.s.hht.att.thtts.
```

**Figure 1.** Continued where this domain is present. A list of 18 proteins comprising this domain is shown in Table 1B and some proteins, as indicated, contain more than one domain. The proteins comprising this domain are described as either conserved, hypothetical or predicted proteins and correspond to the *M. acetivorans, M. mazei* or *M. barkeri* genomes. One of the proteins is disaggregatase from *M. mazei*. Four distinct regions within this domain are characterized by conserved sequence motifs; DNRLRE, LSLxWYYP, YENTGFLIK, AFYS. For the sake of simplicity, we refer to this 200 aa region as the DNRLRE domain. The pairwise percentage sequence identities corresponding to the DNRLRE domain varies (range 42–100%). The consensus secondary structure predicted for

Copyright © 2004 John Wiley & Sons, Ltd. 

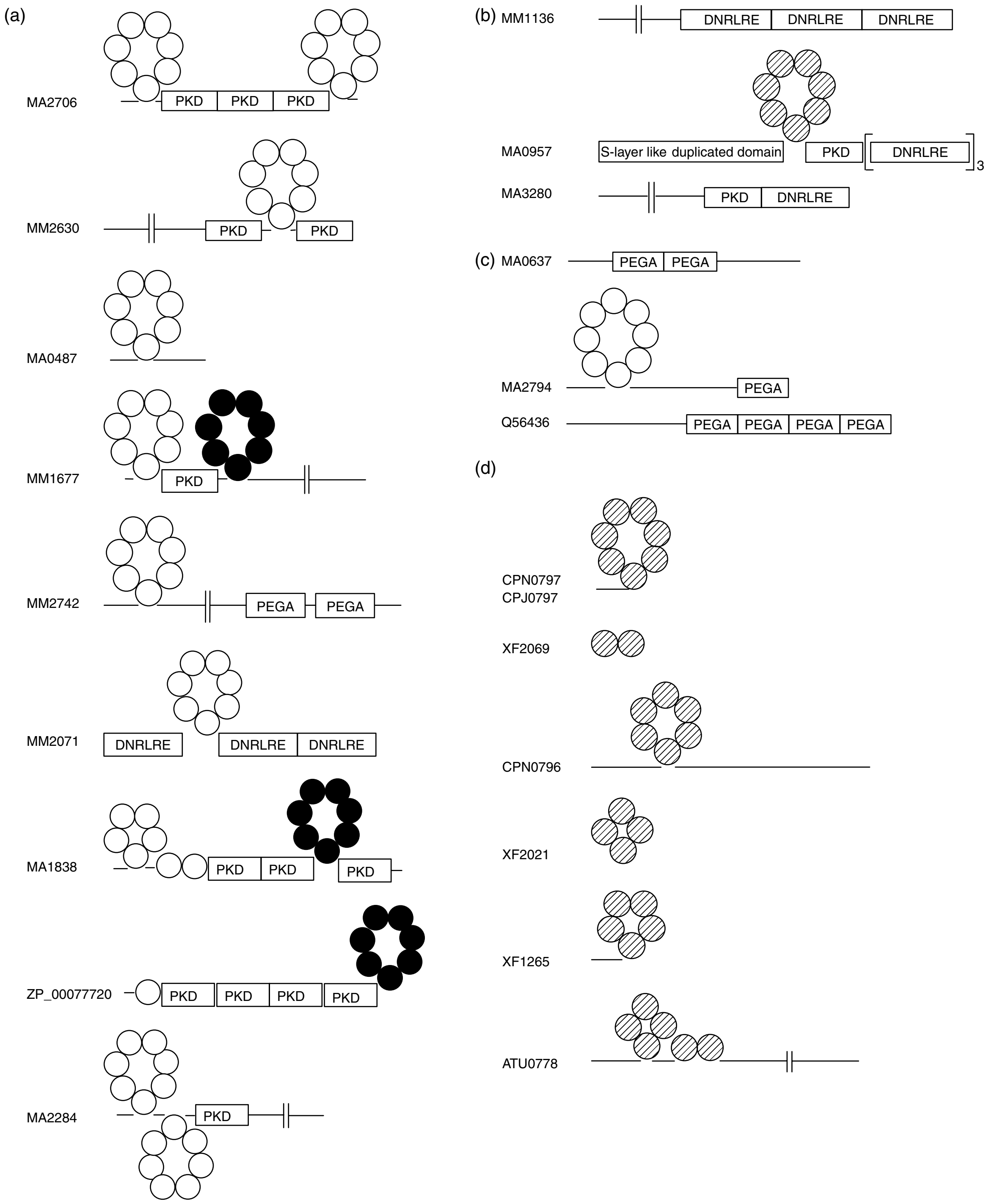

**Figure 2.** (A) ◯ represents a single RIVW repeat; ● represents a single YVTN (or AB) repeat; DNRLRE; PKD; PEGA represent the corresponding domains. (B) ⊘ represents LGxL repeat; (E) ⊕ represents LVIVD repeat; [||||] represents the LGFP repeat and (F) Ag 85-like domain represents a 285 amino acid residue antigen 85-like protein; PGRP represents peptidoglycan recognition protein

Copyright © 2004 John Wiley & Sons, Ltd. 

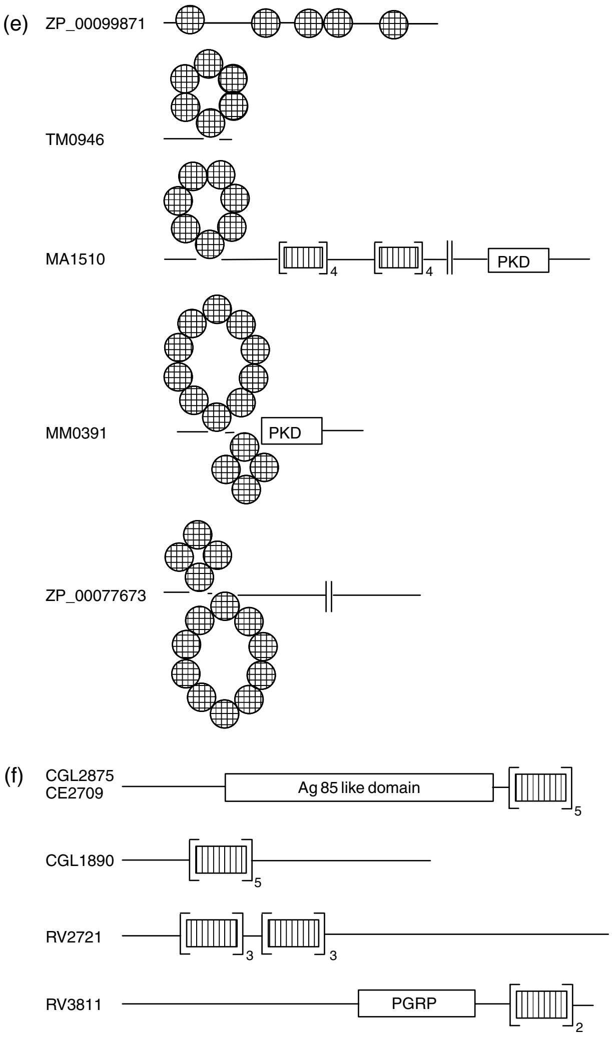

**Figure 2.** Continued this domain suggests mainly $\beta$-strands and two $\alpha$-helices (see Figure 1B). The association of the conserved sequence motifs to regular secondary structure may be inferred from Figure 1B. Further, based on the BLAST sequence analysis corresponding to the region outside the DNRLRE domain, we noticed that some of these proteins also contain other well-characterized regions identified by others earlier, such as PbH1, CADG and TonB-boxC. These are also indicated in Table 1B. For details of these other regions refer the INTERPRO database (Mulder *et al*., 2003).

The schematic representation of the domain architectures that represent these 18 proteins is shown in Figure 2B. These comprise either three DNRLRE domains, as in the protein with gene identifier MM1136 or associated with a PKD domain, S-layer duplicated domain and a LGxL tandem repeat (described later in this work) as in the protein with the gene identifier MA0957. Also, the DNRLRE domain-containing proteins, like the proteins containing RIVW repeats, appears to be specific only to the organisms of genus *Methanosarcina* and may be involved in mediating specific cell functions on the cell surface.

### 70 aa residues PEGA domain

RADAR analysis of the protein with gene identifier MM2742 (described as a hypothetical protein

Copyright © 2004 John Wiley & Sons, Ltd. 

in the *M. acetivorans* genome) indicated a 70 aa residue region that is present as two copies in addition to the RIVW tandem repeats, as shown in Figure 2A. These two 70 aa residue regions share 41% sequence identity. The BLAST program searches against the database with query sequence corresponding to the aa sequence in the region 661–723 positions in MM2742 identified other proteins. The list of proteins containing this domain is shown in Table 1C. As can be seen, the domain is observed in *Thermus thermophilus, Thermotoga maritima, Leptospira interrogans* and *M. acetivorans* and described as either hypothetical or S-layer like proteins. The multiple sequence alignment corresponding to this domain identified two characteristic sequence motifs; PEGA and LxxxG, where x is any aa residue. This is shown in Figure 1C. We refer to this as the PEGA domain. The secondary structure is predicted to comprise essentially $\beta$-strands. Based on the secondary structure predictions, we observed that PEGA sequence corresponds to a loop connecting two $\beta$-strands, whereas LxxxG sequence corresponds to the end of a $\beta$-strand and a portion of a loop connecting another $\beta$-strand. The pairwise percentage sequence identities vary (14–46%). The domain architecture representing proteins containing PEGA domain is shown in Figure 2C. The protein with gene identifier MA2794 is also associated with eight RIVW tandem repeats. By inference, we propose that the two hypothetical proteins with gene identifiers, MM2742 in *M. mazei* and MA2794 in *M. acetivorans* (see Table 1C), which comprise the PEGA domain in addition to the RIVW repeats may also be S-layer like proteins.

### 45 aa residues LGxL repeat

The 1196 aa residues containing protein corresponding to gene identifier MA0957 in *M. acetivorans* comprises a 45 aa residues repeat. Each repeat corresponds to the following conserved sequence motifs; LGxL, VVG, HA distributed along the repeat sequence. For simplicity we refer to these as the LGxL repeats. These repeats are present in addition to the PKD domain, S-layer-like duplicated domain and three DNRLRE domains as shown in Figure 2B. The sequence homology shared between this LGxL repeats in MA0957 varies (30–76%). The search of the database with BLAST program using the query sequence corresponding to the region 222–264 in MA0957 identified several 'hypothetical' proteins containing this repeat region from organisms such as *Anabena* sp, *Chalmydia pneumoniae, Xylella fastidiosa* and *Agrobacterium tumefaciens*. The list of 13 proteins containing this repeat is shown in Table 1D. The length of proteins identified varied (94–1196 aa residues). Each protein represented a variable number of tandem repeats. We observed that proteins containing the LGxL repeat seem not to be associated with the repeats or the domains described until now (see Figure 2D), except the protein corresponding to the gene identifier MA0957 (see Figure 2B). The multiple sequence alignment corresponding to this repeat is shown in Figure 1D. The pairwise percentage sequence identities between sequences corresponding to LGxL repeats vary (6–68%). The secondary structure is predicted to comprise four $\beta$-strands and the conserved sequence motifs described above are associated with $\beta$-strands (see Figure 1D). The representative domain architecture corresponding to proteins comprising the LGxL tandem repeats (also likely to be associated as a $\beta$-propeller fold) is shown in Figure 2D.

### 42 aa residues LVIVD repeats

The protein corresponding to the gene identifier MA1510, comprising 2115 aa residues in *M. acetivorans* and described as a hypothetical protein, contains approximately 42 aa residues repeat regions. Each repeat present in tandem is associated with YAYV, LVIVD sequence motifs. We refer to these as the LVIVD repeats. The sequence identities vary (17–72%). In addition, the RADAR program also identified another repeat region comprising ~54 aa residues in MA1510, which is discussed later. The BLAST searches corresponding to the LVIVD repeats (region 170–210 in MA1510) identified a number of proteins from various organisms such as *M. mazei, M. barkeri, Thermotoga maritime, Microbulbifer degradans, Magnetococcus* MC-1, *Desulfitobacterium hafniense* that are classified as hypothetical proteins. The list of eight proteins containing the 42 aa residue LVIVD repeats and the number of repeats observed in the protein is indicated in Table 1E. The pairwise percentage sequence identities in this case vary (10–83%). The secondary structure corresponding

Copyright © 2004 John Wiley & Sons, Ltd. 

to the LVIVD repeat is predicted to comprise four $\beta$-strands, as shown in Figure 1E and the four $\beta$-strands may be associated as a $\beta$-propeller. The representative domain architectures corresponding to proteins containing this repeat are shown in Figure 2E. Some of the LVIVD repeat-containing proteins may also be associated with PKD domain or the LGFP repeat (discussed below).

### 54 aa residues LGFP repeat

The protein corresponding to the gene identifier MA1510 contains a 54 aa residues repeat with a conserved L-G-x-P-x(7)-D-G sequence motif. For simplicity we refer to this as the LGFP repeat. Four such repeats are present in tandem and there are two distinct regions along the sequence associated with the LGFP tandem repeats. The two regions are located between the well-characterized PKD domain and LVIVD tandem repeats. The BLAST searches of the LGFP sequence (corresponding to the 603–654 aa residue region in MA1510) identified the LGFP repeats in several proteins from different genomes, e.g. *Corynebacterium glutamicum, C. efficiens, M. tuberculosis, M. leprae, M. paratuberculosis, Deinococcus radiodurans*. Table 1F indicates the 13 proteins comprising the LGFP tandem repeats and the number of times these are observed. Once again, many proteins described as 'hypothetical' in the SWall database are observed. The *Mycobacterium tuberculosis* protein Rv3811 is a cell surface protein (CSP) and the protein from the bacterial species *D. radiodurans,* DR1115, is a S-layer-like array related protein. Two proteins, CGL2875 and CE2709, are PS1 proteins of *C. glutamicum* and *C. efficiens*, respectively. The multiple sequence alignment corresponding to the repeat shown in Figure 1F suggests that the aa conservation is more towards the N-terminal half of the repeat region. The pairwise percentage sequence identities vary (15–98%). The secondary structure is predicted to comprise two $\beta$-strands and one $\alpha$-helix (see Figure 1F). The representative domain architecture for proteins comprising this repeat is shown in Figure 2F.

In another context, we know that the PS1 and PS2 are two major secretory proteins in *C. glutamicum* genome (Joliff *et al*., 1992). Freeze-fracture electron microscopy studies of *C. glutamicum* indicated the presence of ordered arrays on its surface associated with the PS2 protein (Chami *et al*., 1995). The PS1 protein corresponding to the gene identifier CGL2875 (comprising 657 aa residues) in *C. glutamicum* is encoded by the *csp1* gene and is also associated with the cell wall. CGL2875 has a N-terminal region that is similar to *M. tuberculosis* antigen 85 complex, which functions as a mycolyl transferase that catalyses the transfer of mycolic acid to arabinogalactan and trehalose monomycolate (Puech *et al*., 2000). It has been shown that the N-terminal region (of CGL2875) possess the mycolyl transferase activity and the C-terminal region is not required for this activity (Puech *et al*., 2000). The five LGFP tandem repeats identified by us correspond to the C-terminal region in *C. glutamicum* (gene identifier CGL2875) and *C. efficiens* (gene identifier CE2709), as shown in Table 1F and in the corresponding Figure 2F. We therefore hypothesize that the PS1 proteins in *Corynebacterium* (CGL2875 and CE2709), when associated with the cell wall, may be anchored via the LGFP tandem repeats that may be important for maintaining cell wall integrity. Experimental evidence to our hypothesis comes from the recent work of Brand *et al*. (2003) who demonstrated that the deletion of CGL2875 protein resulted in a 10-fold increase in the cell volume of the organism and inferred the corresponding protein's involvment in the cell shape formation.

### Conclusions

A systematic analysis combining automated tools and manual evaluation identified four novel tandem repeats and two domains corresponding to the cell surface proteins in *M. acetivorans*. Further database searches corresponding to these newly identified tandem repeats and domains identified several other proteins, some of as-yet uncharacterized function, thereby associating cell surface-like properties to such proteins. The RIVW repeats and DNRLRE domain specific to the genus *Methanosarcina* may be responsible for structural organization and function specific to the cell wall in *Methanosarcina*. The repeats and domains identified in the present work that are common to several other organisms may mediate some important cellular function in proteins specific to archaeal and bacterial species. The proteins comprising LGxL, LVIVD and LGFP repeats amongst other repeats analysed may be associated with lower than 15%

Copyright © 2004 John Wiley & Sons, Ltd. 

sequence identity. The RIVW, LGxL and LVIVD repeats are predicted to comprise four $\beta$-strands per repeat, and the proteins comprising seven such tandem repeats may be associated with a $\beta$-propeller fold reminiscent of the cell surface proteins comprising the tandem AB repeats associated with a $\beta$-propeller fold. The identification of novel repeats and domains corresponding to cell surface proteins from various organisms may be useful for annotation.

### Acknowledgements

S.A. thanks CSIR, New Delhi, India, for a Research fellowship. L.G. thanks DST, Government of India, for a Young Scientist Fast Track Fellowship. The authors would like to thank the referees for their invaluable comments.

### References

Adindla S, Guruprasad L. 2003. Sequence analysis corresponding to the PPE, PE proteins in *Mycobacterium tuberculosis* and other genomes. *J Biol Sci* **28**: 169–179.

Altschul SF, Gish W, Miller W, Myers EW, Lipman DJ. 1990. Basic local alignment search tool. *J Mol Biol* **215**: 403–410.

Altschul SF, Madden TL, Schäffer AA, *et al.* 1997. Gapped BLAST and PSI-BLAST: a new generation of protein database search programs. *Nucleic Acids Res* **25**: 3389–3402.

Andrade MA, Ponting C, Gibson T, Bork P. 2000. Identification of protein repeats and statistical significance of sequence comparisons. *J Mol Biol* **298**: 521–537.

Andrade MA, Perez-Iratxeta C, Ponting CP. 2001. Protein repeats: structures, functions, and evolution. *J Struct Biol* **134**: 117–131.

Andrade MA, Ciccarelli FD, Perez-Iratxeta C, Bork P. 2002. NEAT: a domain duplicated in genes near the components of a putative $Fe^{3+}$ siderophore transporter from Gram-positive pathogenic bacteria. *Genome Biol* **3**: 0047.1–0047.5.

Bateman A, Birney E, Cerruti L, *et al.* 2002. The Pfam Protein Families Database. *Nucleic Acids Res* **30**: 276–280.

Beveridge TJ, Graham LL. 1991. Surface layers of bacteria. *Microbiol Rev* **55**: 684–705.

Beveridge TJ. 1994. Bacterial S-layers. *Curr Opin Struct Biol* **4**: 204–212.

Brand S, Niehaus K, Puhler A, Kalinowski J. 2003. Identification and functional analysis of six mycolyltransferase genes of *Corynebacterium glutamicum* ATCC 13032: the genes cop1, cmt1, and cmt2 can replace each other in the synthesis of trehalose dicorynomycolate, a component of the mycolic acid layer of the cell envelope. *Arch Microbiol* **180**: 33–44.

Bycroft M, Bateman A, Clarke J, *et al.* 1999. The structure of a PKD domain from polycystin-1: implications for polycystic kidney disease. *EMBO J* **18**: 297–305.

Chami M, Bayan N, Dedieu J, *et al.* 1995. Organization of the outer layers of the cell envelope of *Corynebacterium glutamicum*: a combined freeze-etch electron microscopy and biochemical study. *Biol Cell* **83**: 219–229.

Cossart P, Jonquieres R. 2000. Sortase, a universal target for therapeutic agents against Gram-positive bacteria? *Proc Natl Acad Sci USA* **97**: 5013–5015.

Falquet L, Pagni M, Bucher P, *et al.* 2002. The PROSITE database, its status in 2002. *Nucleic Acids Res* **30**: 235–238.

Galagan JE, Nusbaum C, Roy A, *et al.* 2002. The genome of *M. acetivorans* reveals extensive metabolic and physiological diversity. *Genome Res* **12**: 532–542.

Heger A, Holm L. 2000. Rapid automatic detection and alignment of repeats in protein sequences. *Proteins* **41**: 224–237.

Jing H, Takagi J, Liu JH, *et al.* 2002. Archaeal surface layer proteins contain beta propeller, PKD, and $\beta$-helix domains and are related to metazoan cell surface proteins. *Structure (Camb)* **10**: 1453–1464.

Joliff G, Mathieu L, Hahn V, *et al.* 1992. Cloning and nucleotide sequence of the csp1 gene encoding PS1, one of the two major secreted proteins of *Corynebacterium glutamicum*: the deduced N-terminal region of PS1 is similar to the *Mycobacterium* antigen 85 complex. *Mol Microbiol* **6**: 2349–2362.

Kandler O, Konig H. 1993. Cell envelopes of Archaea: structure and chemistry. In *The Biochemistry of Archaea*, Kates M, *et al.* (eds). Elsevier: Amsterdam; 223–259.

Letunic I, Goodstadt L, Dickens NJ, *et al.* 2002. Recent improvements to the SMART domain-based sequence annotation resource. *Nucleic Acids Res* **30**: 242–244.

Lupas A, Englehardt H, Peters J, *et al.* 1994. Domain structure of the *Acetogenium kivui* surface layer revealed by electron crystallography and sequence analysis. *J Bacteriol* **176**: 1224–1233.

Lupas A. 1996. A circular permutation event in the evolution of the SLH domain? *Mol Microbiol* **20**: 897–898.

Mayerhofer LE, Conway de Macario E, Macario AJL. 1995. Conservation and variability in Archaea: Protein antigens with tandem repeats encoded by a cluster of genes with common motifs in *Methanosarcina mazei* S-6. *Gene* **165**: 87–91.

Mesnage S, Fontaine T, Mignot T, *et al.* 2000. Bacterial SLH domains are non-covalently anchored to the cell surface via a conserved mechanism involving polysaccharide pyruvylation. *EMBO J* **19**: 4473–4484.

Mulder NJ, Apweiler R, Attwood TK, *et al.* 2003. The InterPro Database, 2003 brings increased coverage and new features. *Nucleic Acids Res* **31**: 315–318.

Navarre WW, Schneewind O. 1999. Surface proteins of Gram-positive bacteria and mechanisms of their targeting to the cell wall envelope. *Microbiol Mol Biol Rev* **63**: 174–229.

Puech V, Bayan N, Salim K, Leblon G, Daffe M. 2000. Characterization of the *in vivo* acceptors of the mycoloyl residues transferred by the corynebacterial PS1 and the related mycobacterial antigens 85. *Mol Microbiol* **35**: 1026–1041.

Rost B, Sander C, Schneider R. 1994. PHD — an automatic mail server for protein secondary structure prediction. *CABIOS* **10**: 53–60.

Schaftenaar G, Cuelenaere K, Noordik JH, Etzold T. 1996. A Tcl-based SRS v. 4 interface. *Comput Appl Biosci* **12**: 151–155.

Sleytr UB, Messner P, Pum D, *et al.* (eds). 1996. *Crystalline Bacterial Cell Surface Proteins*. Academic Press: London.

Thompson JD, Higgins DG, Gibson TJ. 1994. CLUSTAL W: improving the sensitivity of progressive multiple sequence alignment through sequence weighting, position-specific gap penalties and weight matrix choice. *Nucleic Acids Res* **22**: 4673–4680.

Copyright © 2004 John Wiley & Sons, Ltd. 
